# Serological evidence of *Toxoplasma gondii* infection as potential risk for the development of lepromatous leprosy in an endemic area for both neglected tropical diseases in Brazil

**DOI:** 10.1186/s40249-020-0636-3

**Published:** 2020-02-13

**Authors:** Luciana Regina Pereira Oliveira, Lívia Mattos Martins, Rebeka da Conceição Souza, Yuri Scheidegger de Castro, Letícia Silva Nascimento, Juliana Azevedo da Silva, Edilbert Pellegrini Nahn Junior, Wilmar Dias da Silva, Alba Lucínia Peixoto-Rangel

**Affiliations:** 1 Laboratory of Recognition Biology, Center of Biosciences and Biotechnology, State University of Northern Rio de Janeiro, Campos dos Goytacazes, RJ Brazil; 2 Campos Medical School, Campos dos Goytacazes, RJ Brazil; 3 Laboratory of Immunochemistry, Butantan Institute, São Paulo, SP Brazil

**Keywords:** Leprosy, Toxoplasmosis, Coinfection, Co-immunomodulation, Immunoglobulins, Immune response

## Abstract

**Background:**

*Mycobacterium leprae* and *Toxoplasma gondii* infections are both neglected tropical diseases highly prevalent in Brazil. Infection with certain parasite species can significantly alter susceptibility to other important pathogens, and/or influence the development of pathology. Here we investigated the possible influence of *M. leprae*/*T. gondii* co-parasitism on the manifestation of leprosy and its clinical forms.

**Methods:**

Participants (*n* = 291) were recruited in Campos dos Goytacazes city, Rio de Janeiro state, southeast Brazil, from August 2015 to December 2019 and clinically diagnosed for leprosy. Participants were selected based on the presence (patients) or absence (healthy controls) of the leprosy disease. Contacts of patients were also recruited for this study. Serum samples from patients (*n* = 199) with leprosy, contacts (*n* = 40) and healthy controls (*n* = 52) were investigated for levels of IgM and IgG anti-phenolic glycolipid-1 (PGL-1) by ELISA. Additionally, IgG antibody against soluble *Toxoplasma* antigen (STAg) was measured in sera samples from leprosy patients, contacts and healthy controls for *Toxoplasma gondii* serology by ELISA. Anti-PGL-1 IgG and IgM levels were compared using one-way ANOVA Kruskal-Wallis or Mann-Whitney, while Spearman test was used to correlate levels of IgG anti-STAg and IgM/IgG anti-PGL-1 from seropositive and seronegative individuals for *T. gondii* infection. The risk of *T. gondii* infection for leprosy disease was assessed using Fisher’s test.

**Results:**

Levels of IgM anti-PGL-1 antibodies were significantly higher in multibacillary (MB) patients compared to paucibacillary (PB) patients (*P* = 0.0068). Higher IgM and IgG levels anti-PGL-1 were detected in patients with the lepromatous forms. The serologic prevalence for *T. gondii* infection was 74.9%. We detected increased anti-STAg antibody levels in leprosy patients (79.4%), reaching 88.8% within those with lepromatous form of this disease. The leprosy risk increase in *T. gondii* seropositive individuals was two-fold (odds ratio [*OR*] = 2.055; 95% confidence intervals [95% *CI*]: 1.18–3.51) higher than those seronegative, and considering the lepromatous leprosy risk this increase was even dramatic (*OR* = 4.33; 95% *CI*: 1.76–9.69) in *T. gondii* seropositive individuals. Moreover the leprosy risk in *T. gondii* seropositive individuals was weakly correlated to the levels of IgG anti-STAg and IgM/IgG anti-PGL-1.

**Conclusions:**

Altogether, our results suggest that *T. gondii* infection may exert immunomodulatory properties that influence to the susceptibility of leprosy, mainly on its more severe clinical form. A better understanding of parasite immunomodulation can ultimately contribute to the development of medical applications.

## Background

Leprosy, caused by *Mycobacterium leprae*, is an infectious disease that affects the skin, peripheral nerves and presents a clinical-pathological spectrum based on the host immune response [1]. By the latest estimates available, leprosy still occurs in Brazil in high levels having been detected around 26 875 new cases in 2017 [2]. Concurrent parasitic infections are common among individuals living under poor sanitary conditions in developing countries. Parasites have evoked a wide range of mechanisms to evade and/or manipulate the host’s immune response and establish infection [3–6]. An increasing number of evidence shows that co-infection by pathogens might alter susceptibility to other important pathogens, and/or influence vaccine efficacy through their effects on host immune responsiveness. Soil-transmitted helminth infections have been show to play a role in the progression of leprosy to the more severe clinical type and the occurrence of type 2 reaction in Indonesian individuals [7].

In Brazil, depending on the region studied, the prevalence of *Toxoplasma gondii* infection in adults can range from 50 to 80% [8]. Epidemiological studies in Campos dos Goytacazes-RJ, Brazil presented a high *T. gondii* seroprevalence, reaching even 84% of the low income population [9]. Drinking water has been determined as the main risk factor for *T. gondii* infection for individuals living under poor sanitary conditions in the same studied area [9]. In a previous report, we have shown the immune response against *T. gondii*, in individuals co-infected with *Ascaris lumbricoides*, was marked by low secreted levels of IL-10, IL-4, IL-5 and TGF-β and low levels of IgE against *A. lumbricoides*, compared to groups not infected by *A. lumbricoides*, favoring helminth adaptation in the host, since the specific protective immune response is down-regulated [10]. Co-infected individuals with less severe ocular toxoplasmic lesions were found having elevated levels of IFN-γ (Th1) and IL-13 (Th2). Both cytokines may limit *T. gondii* growth and control the inflammatory response, which would result in better adaptation of *T. gondii* in the host. Thus, it has been proposed that co-evolution may drive parasite products to modulate the host immune response for better adaptation to both parasites [10]. Krahenbuhl et al. [11] demonstrated that previous infection of mice with *T. gondii* provided protection against challenge with *M. leprae* in the footpad of these animals. Subsequently, it was shown that macrophages obtained from footpad granulomas *M. leprae*-infected athymic (*nu*/*nu*) mice were defective in responding to macrophage activation signals, such as IFN-γ [12]. High levels of anti-*T. gondii* antibodies were detected in leprosy patients sera from Pakistan [13]. Lepromatous leprosy and mucosal leshmaniasis, two opposite polar forms of these diseases, were observed in peripheral blood mononuclear cells culture from patients co-infected, which during active leprosy, the *M. leprae* antigens induced suppression of the IFN-у response to *Leishmania braziliensis* antigen, and this suppression was abolished by IL-10 neutralization. Moreover this suppressive effect was lost after the cure of leprosy and the disappearance of this effect was accompanied by worsening of mucosal leishmaniasis lesions. The results indicated that leprosy induced an IL-10-mediated regulatory response that could have controlled the mucosal leishmaniasis immunopathology, demonstrating that, in the context of this co-infection, the immune response to one pathogen may influence the immune response of the other pathogen and to the clinical course of the infection caused by it [14].

Epidemiological characteristics of leprosy and toxoplasmosis, raise our interest in better understanding the possible influence of *T. gondii* infection on the outcome of *M. leprae* disease, both endemic in Brazil. Because leprosy primarily affects populations living in poverty environments, *T. gondii* infection can commonly be found as comorbidity in leprosy patients. In this paper, we evaluate the influence of *M. leprae*-*T. gondii* co-parasitism in the manifestation of leprosy and its clinical forms.

## Methods

### Subjects

Cases and healthy controls were recruited in Campos dos Goytacazes city, Rio de Janeiro state, southeast Brazil (21°45′15“ S and 41°19’28” W). One hundred and ninety-nine leprosy patients were selected at the Hansen Health Program from Campos dos Goytacazes Health Secretariat, which is considered a reference center for treatment of this disease. Forty household contacts were also recruited for this study. All participants were clinically diagnosed according to the Brazilian’s Ministry of Health Guidelines and patient’s diagnosis was complemented with bacilloscopy of suspected tissue lesions. Healthy controls consisted of 52 unrelated individuals recruited from the local blood bank (hemocenter) (Table 1). Leprosy patients (LP), household contacts (CT) and healthy control (HC) were from the same geographical area. Leprosy patients were grouped according to the World Health Organization (WHO) classification [15] in multibacillary (MB) or paucibacillary (PB) leprosy and Madrid classification [16] in lepromatous leprosy (LL), dimorph leprosy (DL), indeterminate leprosy (IDL), and tuberculoid leprosy (TL) for the analysis (Table 1). The informed consent (written) was obtained from all participants and the study was approved by the local Medical Ethics Committee (CAEE No. 32510914.7.0000.5244). Sera samples were obtained by centrifugation of fresh whole blood and stored at -20 °C until used.

**Table 1:** Study population

	Clinical groups (OMS)	Clinical groups (Madrid)	*N*	Age Mean ± SD (Range)	Gender Female/Male(%)
Leprosy	Multibacillary	Lepromatous	70	45.4 ± 18.6 (16–91)	19.0/81.0
		Dimorph	66	50.4 ± 20.5 (6–93)	32.0/68.0
	Paucibacillary	Indeterminate	15	30.5 ± 13.3 (11–51)	53.0/47.0
		Tuberculoid	48	42.4 ± 19.5 (13–86)	52.0/32.0
Healthy controls			52	34.6 ± 11.3 (21–60)	23.0/77.0
Household contacts			40	46.9 ± 16.3 (9–71)	65.0/35.0
			40	(9–71)	65.0/35.0
Total			291		

### Soluble toxoplasma antigen preparation

Tachyzoite forms of *Toxoplasma gondii* parasites RH strain, maintained in female swiss mice, of approximately, 3–4 weeks old were recovered 2–3 days after infection, for soluble *Toxoplasma* antigen (STAg). The mice were maintained under suitable ethical conditions, in ventilated cages with free access to water and food, in agreement with international recommendations [17]. STAg were prepared as following: mice peritoneal fluid containing *T. gondii* tachyzoit forms were centrifuged at 100 × *g* for 5 min; the supernatant was centrifuged again at 913 × *g* for 30 min at 4 °C. A small quantity of PBS was added to the parasite sediment and an aliquot of this suspension was removed and diluted 1:100 for counting in a Newbauer chamber. Approximately, 2.5 × 10^8^ parasites per milliliter were exposed to six pulses, of 30 s each, in ice, using ultrasound equipment (Branson–Sonifier 150) and centrifuged at 900 × *g* for 20 min. The supernatant was transferred to another tube and centrifuged again at 10000 × *g* for 10 min. Protein concentration in the supernatant (STAg) was determined by Lowry method [18], and the antigen was then stored at -20 °C until use.

### Phenolic Glycolipid-1 (PGL-1) ELISA

IgG and IgM anti-PGL-1 antibodies were detected by enzyme-linked immunosorbent assay (ELISA) as previously described by Bazan-Furini [19], with some modifications. PGL-1 was coated onto high-affinity polystyrene Nunc™ *MaxiSorp*™ flat-bottom 96 well *plates* (Thermo Scientific, Massachusetts, USA) using 2.0 μg/ml per well in 100 μl of 0.1 mol/L sodium carbonate/bicarbonate pH 9.6 (i.e. coating buffer) at 4 °C overnight. After discarding wells content, sera samples from patients, contacts and healthy controls, diluted of 1:100 in 100 μl of dilution buffer (15 mmol/L Tris pH 7.5 buffer with 0.05% Tween 20, containing 5% bovine serum albumin [BSA]), were added to the wells and incubated for 1 h at 37 °C. Next, sera were discarded and 100 μl of horseradish peroxidase (HRP) conjugated IgG or IgM (Southern Biotech, Alabama, USA) anti-human antibody diluted 1:1000 and 1:600, respectively, in dilution buffer, were added and incubated for 1 h at 37 °C. The plates were washed four times using 200 μl of wash buffer (15 mmol/L Tris pH 7.5 buffer with 0.05% Tween 20) and then 100 μl of a freshly prepared substrate solution {28 mmol/L citric acid, 48 mmol/L dehydrated sodium phosphate, 1 mg/ml ABTS (2,2-azino-bis [3-ethylbenz-thiazoline-G-sulfonic acid])}, and 0.003% H_2_O_2_ was added for color development. The plates were incubated for 20 min (IgG) and for 3 min (IgM). For IgM test, the reaction was stopped with the addition of 100 μl of 15 mmol/L Tris pH 7.5 buffer with 0.05% Tween 20, containing 1% sodium dodecyl sulfate (SDS) and 0.1% azide. Absorbance was determined at a wavelength of 405 nm in ELISA microplate reader (VersaMax™ Tunable Microplate, VWR International, Pensilvânia, USA).

### ELISA for detection of anti-soluble toxoplasma antigen (STAg) antibodies

A 96-well microtiter plates (Nunc™ *MaxiSorp*™) were coated with 100 μl of 0.1 mol/L bicarbonate buffer, pH 9.6, containing STAg (10 μg/ml), for 18–20 h at 4 °C as previously described by Carvalho et al. [20], with some modifications. The plates were three times washed with PBST (PBS 1×; 0.05% Tween 20). Then, 100 μl of blocking buffer (PBST, 1% BSA) was added in each well and incubated for 30 min at 4 °C. The plates were washed three times and samples (including positive and negative controls) were diluted 1:1000 in diluent buffer (PBST; 0.5% BSA) and added 100 μl to the wells in duplicated. The samples were incubated for 1 h at room temperature. After washing, antibodies IgG HRP anti-human (Southern Biotech, Alabama, USA) were diluted 1:1000 in diluent buffer and 100 μl added to each well and incubated for 1 h at room temperature. The plates were washed and 100 μl of a freshly prepared substrate solution (28 mmol/L citric acid, 48 mmol/L dehydrated sodium phosphate, 1 mg/ml ABTS and 0.003% H_2_O_2_) was added for color development. The reaction was stopped by the addition of 30 μl of citric acid (0.2 mol/L) and plates read at 405 nm in a reader (VersaMax™ Tunable Microplate, VWR International, Pensilvânia, USA).

The cut-off point of the test was calculated by the mean of negative controls plus three times the standard deviation of these samples, where values below or equal the cut-off were considered negative, and values above the cutoff were considered positive.

### Statistical analysis

Serum levels of anti-PGL-1 IgG and IGM antibodies were compared using GraphPad Prism v.6 Software (GraphPad Software, La Jolla, CA), to perform intra and intergroup statistical analyzes applying the Kruskal-Wallis test, followed by Dunn’s test (post test) for comparisons among three or more groups. While the Mann-Whitney test was used for comparison between two groups. Correlation test (Spearman) between levels of IgG anti-STAg and IgM/IgG anti-PGL-1 from seropositive and seronegative individuals for *T. gondii* infection were also done using GraphPad Prism v.6 Software. Risk analyzes were also performed using Prism with contingency tables and Fisher’s test application. All analyzes considered significant *P*-values < 0.05.

## Results

### Detection of IgM and IgG anti-PGL-1 in leprosy patients, contacts and healthy controls

**Fig. 1:**
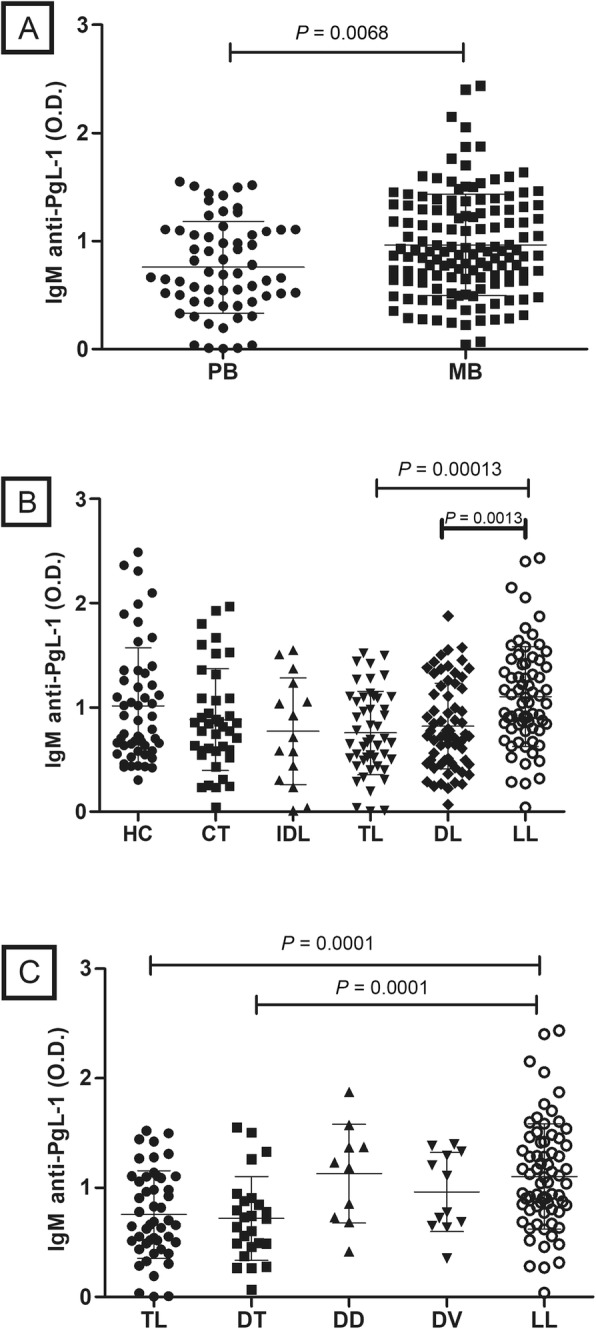
Detection of anti-PGL-1 IgM titers in patients, contacts and control samples. **A** WHO classification (PB- paucibacillary, MB- multibacillary); **B** Madrid classification (HC- healthy control, CT- contacts, TL- tuberculoid leprosy, IDL- indeterminate leprosy, DL- dimorph leprosy and LL- lepromatous leprosy); and **C** Ridley e Jopling (TL- tuberculoid leprosy, DT- dimorph tuberculoid, DD- dimorph dimorph, DV- dimorph lepromatous, LL- lepromatous leprosy

Levels of IgM anti-PGL-1 antibodies were significantly higher in multibacillary (MB) patients compared to paucibacillary (PB) patients (*P* = 0.0068) (Fig. 1A). Considering the Madrid classification, lepromatous leprosy (LL) patients had significantly higher IgM levels compared to tuberculoid (TL) (*P* = 0.0013) and dimorph (DL) (*P* = 0.0013) patients (Fig. 1B). Patients presenting dimorph dimorph (DD) and dimorph lepromatous (DV) were more similar to LL patients, and this last one had significantly higher IgM levels than tuberculoid (TL) and dimorph tuberculoid (DT) patients (Fig. 1C).

**Fig. 2:**
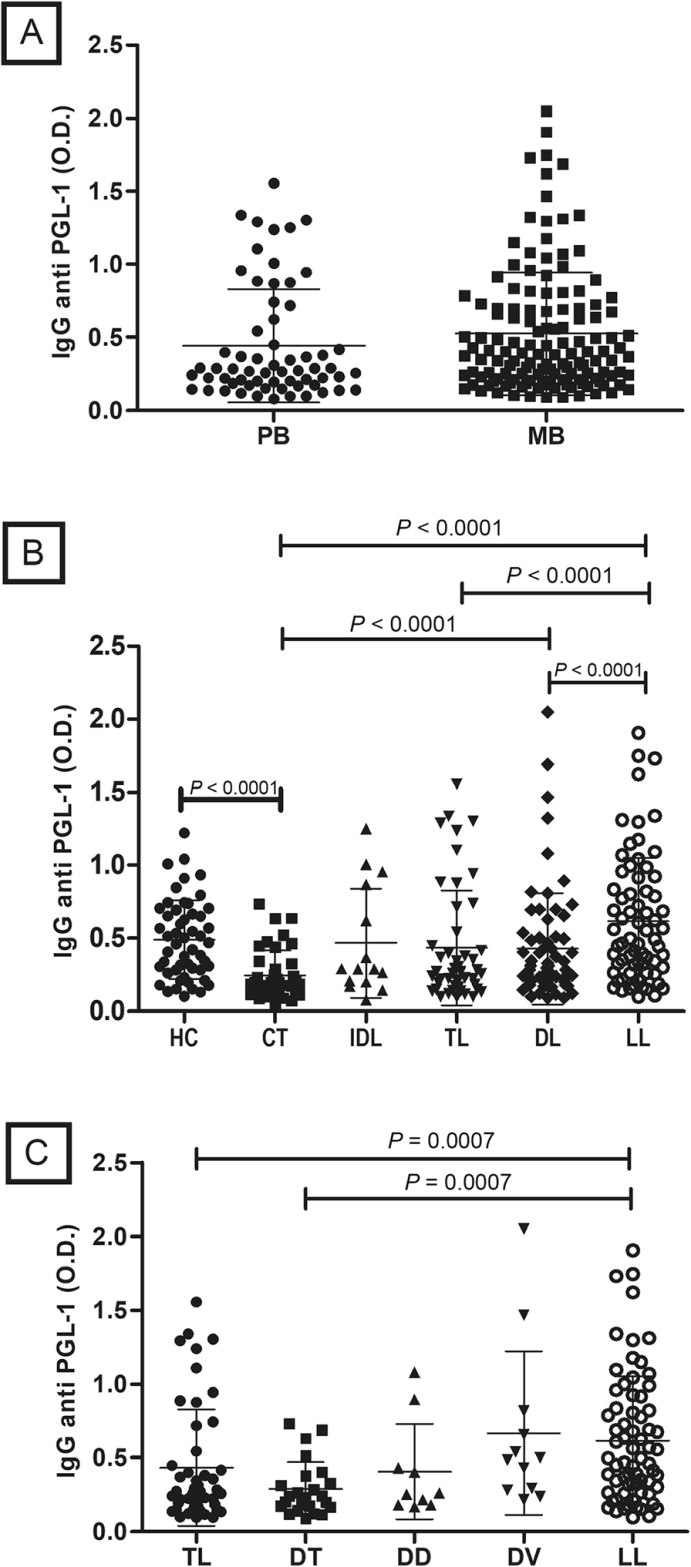
Detection of IgG anti-PGL-1 titers in patients, contacts and control samples. **A** WHO classification (PB- paucibacillary, MB- multibacillary); **B** Madrid classification (HC- healthy control, CT- contacts, TL- tuberculoid leprosy, IDL- indeterminate leprosy, DL- dimorph leprosy and LL- lepromatous leprosy); and **C** Ridley e Jopling (TL- tuberculoid leprosy, DT- dimorph tuberculoid, DD- dimorph dimorph, DV- dimorph lepromatous, LL- lepromatous leprosy

Contrary to IgM analysis, IgG anti-PGL-1 antibody levels were not statistically different between PB and MB patients (Fig. 2A). However, LL patients presented IgG anti-PGL-1 levels increased in relation to DL, TL and CT individuals. Similarly, HC also presented higher levels of IgG anti-PGL-1 than CT patients (Fig. 2B). In Fig.2C it is observed increased titers of IgG anti-PGL-1 in LL patients in relation to DT and TL patients.

### Serological prevalence of *T. gondii* infection in leprosy patients, contacts and healthy controls

IgG anti-STAG was detected in 74.9% of the studied population. In patients with leprosy, the prevalence of *T. gondii* infection was 79.4%. According to the clinical forms, the serological prevalence of *T. gondii* infection was increased in lepromatous leprosy, reaching 88.6%, compared to the others clinical forms (70.8% TL, 73.3% IDL, 77.3% DL), contacts (60%) and healthy controls (69.2%) (Table 2).

**Table 2:** Serological prevalence for *Toxoplasma gondii* infection in leprosy patients, contacts and healthy controls

Individuals	Classification		*n*	Positive IgG anti-*T.gondii, n* (%)	Negative IgG anti-*T.gondii, n* (%)
	WHO	Madrid			
Patients	PB	Indeterminate	15	11 (73.3%)	4 (26.7%)
		Tuberculoid	48	34 (70.8%)	14 (29.2%)
	MB	Dimorph	66	51 (77.3%)	15 (22.7%)
		Lepromatous	70	62 (88.6%)	8 (11.4%)
			199	158 (79.4%)	41 (20.6%)
Controls			52	16 (30.8%)	36 (69.2%)
Contacts			40	24 (60.0%)	16 (40.0%)
Total			291	218 (74.9%)	73 (25.1%)

*MB* Multibacillary, *PB* Paucibacillary, *IgG* Immunoglobulin G

### *Toxoplasma gondii* infection as risk factor for leprosy

Due to the seropositivity for *T. gondii* infection has been increased in lepromatous patients compared to the other clinical forms, Fisher test for risk analyses was conducted in order to verify if *T. gondii* infection would be a risk factor for leprosy development, mainly in its severe form (Table 2). *Toxoplasma gondii* seropositive individuals had the leprosy risk increased in two-fold (odds ratio [*OR*] = 2.055; 95% confidence intervals [95% *CI*]: 1.18–3.51) compared to seronegative. Considering WHO classification, *T. gondii* infection increased the risk to develop multibacillary (MB) leprosy forms in almost three-fold (*OR* = 2.620; 95% *CI*: 1.409–4.874), compared to paucibacillary forms (*OR* = 1.263; 95% *CI*: 0.6355–2.511), which did not present statistical significance (Table 3).

**Table 3:** *Toxoplasma gondii* infection as risk factor for leprosy

	*T. gondii* positive	*T. gondii* negative	*OR*	95% *CI*	*P-*value
Leprosy	158	41	2.055	1.186–3.561	**0.0131**
Not leprosy	60	32			
MB	113	23	2.620	1.409–4.874	**0.0026**
Not leprosy	60	32			
PB	45	19	1.263	0.6355–2.511	0.6032
Not leprosy	60	32			
LL	62	8	4.133	1.762–9.694	**0.0008**
Not leprosy	60	32			
TL	34	14	1.295	0.6081–2.759	0.572
Not leprosy	60	32			

*OR* Odds ratio, *CI* Confidence interval, *MB* Multibacillary, *PB* Paucibacillary, *LL* Lepromatous leprosy, *TL* Tuberculoid leprosy, Fisher’s test for risk analyzes

Since *T. gondii* infection has indicated to be a risk factor for MB leprosy development, we assessed if this infection was also a risk factor for leprosy progression to severe clinical forms in Madrid classification. In Table 3 we can see that in deed *T. gondii* infection increase the lepromatous leprosy risk in four-fold (*OR* = 4.133; 95% *CI*: 1.762–9.694) compared to tuberculoid forms that presented no statistical significance (*OR* = 1.295; 95% *CI*: 0.6081–2.759).

**Fig. 3:**
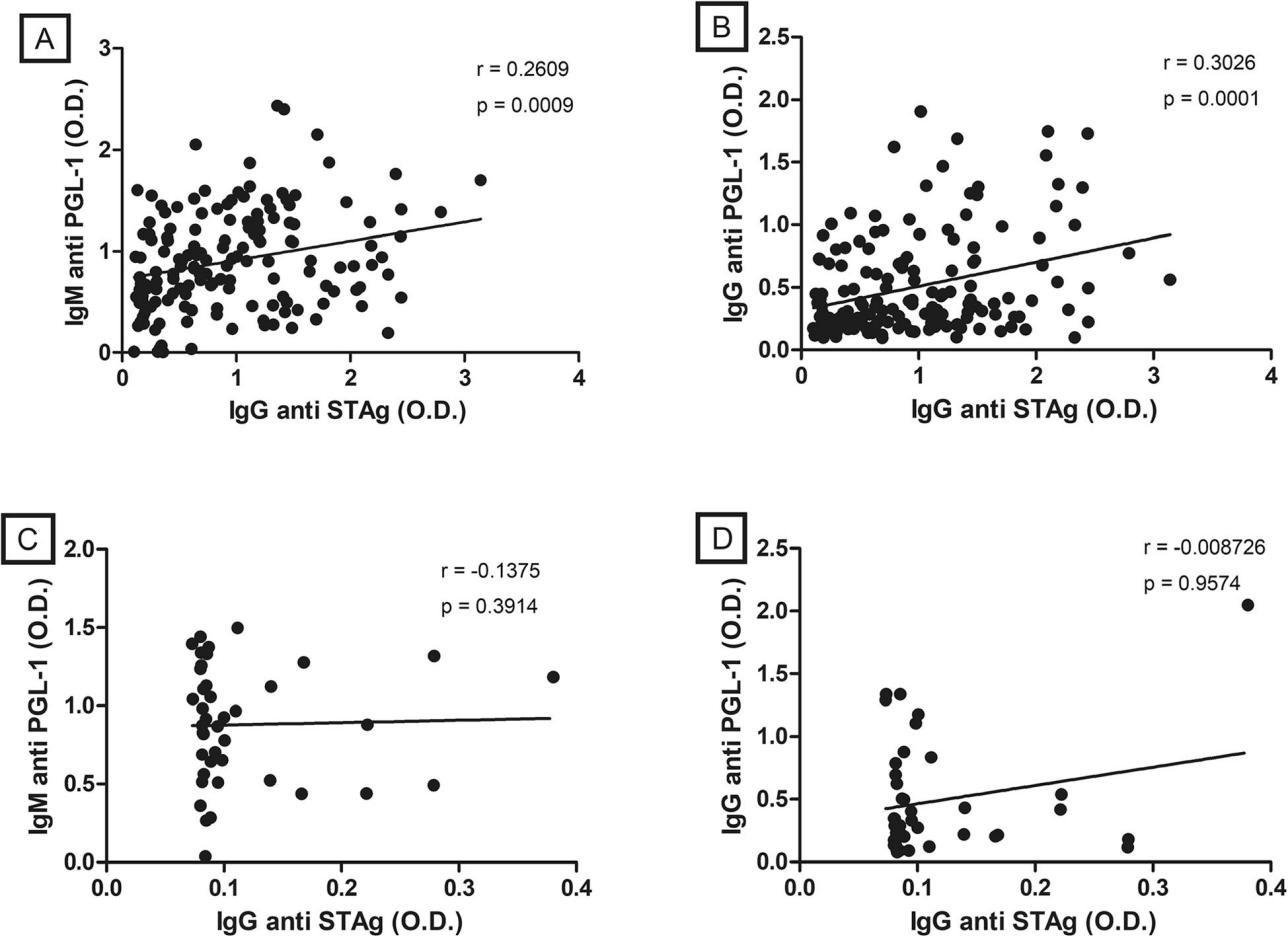
Correlation between IgG anti-STAg and IgM/IgG anti-PGL-1 levels. **a** IgG anti-STAg and IgG anti-PGL-1 levels from *T. gondii* seropositive individuals; **b** IgG anti-STAg and IgM anti-PGL-1 levels from *T. gondii* seropositive individuals; **c** IgG anti-STAg and IgG anti-PGL-1 levels from *T. gondii* seronegative individuals; **d** IgG anti-STAg and IgM anti-PGL-1 levels from *T. gondii* seronegative individuals. STAg: soluble Toxoplasma antigen; PGL-1: phenolic glycolipid 1. Scatter plot was constructed from the raw non-normalized, using GraphPad Software Prism v.6

Finally, to know whether the risk of *T. gondii* infection was associated to the titers of IgG anti-STAg and IgM/IgG anti-PGL-1, we performed the correlation analysis from seropositive and seronegative individuals for *T. gondii* infection (Fig. 3). There was a significant positive correlation between the levels of IgG anti-STAg and both IgM/IgG anti-PGL-1 in *T. gondii* seropositive individuals, but these correlations were weak [IgM (*r* = 0.2609)/ IgG (*r* = 0.3026)] what may mean that antibody levels are not related to the risk of developing leprosy (Fig. 3A and B). We found no correlation between IgG anti-STAg and IgM/IgG anti-PGL-11 from seronegative individuals for *T. gondii* infection (Fig. 3C and D).

## Discussion

Here we have suggested that *T. gondii* infection may exert influence on leprosy susceptibility since the leprosy risk increase in *T. gondii* seropositive individuals was two-fold higher than those seronegative, and considering the lepromatous leprosy risk this increase was even dramatic in *T. gondii* seropositive individuals.

Phenolic glicolipid-1 (PGL-1) is the *M. leprae*-specific antigen [21] and detection of IgM and IgG anti-PGL-1 suggest *M. leprae* infection [22]. IgM anti-PGL-1 is detected at higher levels during the long period of infection. However, IgG and IgA anti-PGL-1 specific antibodies can also be detected [23].

Anti-PGL-1 IgM isotype has been used in searching for infection, but not necessarily for the disease, because detectable IgM levels can be found in both infection and disease, although it has been demonstrated that there is a good correlation between the IgM antibody and bacillary load [24], what could explain the levels of IgM to be higher in multibacillary compared to paucibacillary. Moreover, high levels of IgM have also been associated with increased risk for developing leprosy [25]. Although useful in identifying multibacillary (MB) patients, anti-PGL-1 antibody levels have little value in detecting paucibacillary (PB) patients, since they develop cellular and non-humoral immunity and therefore often have low or no antibody levels. Antibody levels generally increase as the spectrum from tuberculoid disease (TL) progresses to Lepromatous (LL) form [26, 27]. Interestingly, we observed that IgM anti-PGL1 level is a good marker to distinguish tuberculoids from lepromatous, even within dimorph patients. Our study showed IgM anti-PGL-1 antibody levels significantly higher in MB patients and lower in PB, while LL patients had higher antibody levels compared to TL and DL patients. Frota et al. [28] and Fabri et al. [29] had also reported higher serum levels of IgM anti-PGL-1 in MB patients compared to those IgM levels observed in PB patients, corroborating our results.

Many studies have shown anti-PGL-1 IgM serology as a tool for early detection of leprosy in household contacts (CT) of patients with the disease [19, 22, 30]. Several studies have reported that high levels of anti-PGL-1 IgM in CT of leprosy patients were related to increased risk of leprosy development [30–32]. Douglas et al. [33] reported that IgM anti-PGL-1 high titers in CT of multibacillary patients had 7.2-fold greater risk of developing leprosy, and 24-fold higher risk to develop multibacillary leprosy compared to anti-PGL-1 IgM seronegative contacts. We also showed anti-PGL-1 IgM levels increased in CT, but also in healthy controls (HC). According to Calado et al. [34], in endemic areas for leprosy, intra-household and peridomiciliary contacts, there are no significant differences in seropositivity for anti-PGL-1 IgM. Individuals are commonly exposed to *M. leprae* and develop some degree of immune response, producing anti-PGL-1 antibodies [35]. This could explain the high titers of anti-PGL-1 IgM presented in the HC group.

However, not all people exposed to the bacillus who develop anti-PGL-1 antibodies will develop clinical disease [22]. Few serological studies have been performed using anti-PGL-1 IgG [36–39]. The anti-PGL-1 IgM response is uniformly higher than IgG [36]. Several authors have referenced anti-PGL-1 IgM antibody as a parameter for leprosy serology [29, 30, 40, 41]. However, it is also possible anti-PGL-1 IgM can be detected in conditions that lead to polyclonal activation of lymphocytes, such as AIDS and psoriasis, even being detected in non-endemic regions [42]. False positives are known to occur in IgM detection due to the presence of rheumatoid factors [39]. In this context, the presence of IgG antibodies in the serum, reflects the safest form of exposure and re-exposure to the bacillus, since these antibodies represent the existence of memory cells at a secondary response moment to *M. leprae* [38]. Cabral et al. [39] observed that IgG levels in controls were lower than in leprosy patients, but did not differ from contacts. It has been proposed that both anti-PGL-1 IgG and IgM isotypes should be measured because of a high frequency of anti-PGL-1 IgM positivity in negative contact samples for anti-PGL-1 IgG. On the other hand, most IgG positive contacts were also positive for IgM antibodies. Healthy controls presented high levels of IgM and IgG, so we can assume that these individuals were exposed to *M. leprae*. Although in our study we found no statistical difference in anti-PGL-1 IgG antibody levels between MB and PB patients, it has already been shown, by Brett and colleagues [37], that anti-PGL-1 IgG and IgM are higher in LL patients compared to the other leprosy groups. Thus, the results corroborate those in the literature, since LL patients had higher IgG levels compared to other individuals. According to the Ridley and Jopling classification [43] in a previous study, Jadhav et al. [44] also reported lower levels of anti-PGL-1 IgG and IgM antibodies in DT patients and higher in LL patients.

Considering leprosy patients, the seroprevalence for *T. gondii* infection increased to 79.4%, and among leprosy patients the highest prevalence for *T. gondii* infection was detected in LL patients, reaching 88.6%. Rao et al. [45] showed a high incidence of *Toxoplasma* antibody in the lepromatous leprosy group in comparison with control group in an Indian population. These authors supposed this could be related to intimate contact with domestic animals or by ingestion of meat from those animals. A high seroprevalence of *T. gondii* antibodies has also been reported in leishmaniasis caused by either *L. donovani* (65%) or *L. brasiliensis* (16%) and in Chagas disease (27.9%) [46]. All these diseases are caused by pathogens which survive and multiply within the macrophage-monocyte system, indicating that there may be a common cause for such a rise in *T. gondii* antibodies in these diseases. Moreover, detection of high titers of anti-*T. gondii* antibodies in sera of patients with leprosy in Pakistan was believed to be induced by an increase *in T. gondii* load in leprosy due to a transient reactivation of latent *T. gondii* infections, as the antibodies in these leprosy patients were not associated with any sign of eye or lymphatic pathology related to toxoplasmosis [13].

So, we hypothesized *T. gondii* infection as a risk factor for leprosy due to both infectious diseases are associated to poverty conditions and be prevalent in the area of the study. Indeed, seropositivity for *T. gondii* infection increases at two-fold the risk to develop leprosy, and specially the most severe form, lepromatous leprosy. However, the condition of having or not *T. gondii* infection, seems to be a risk factor for leprosy development instead of the titers of IgG anti-STAg and IgM/IgG anti-PGL-1. Contrary to our data, BALB/c mice chronically infected with the intracellular protozoan *T. gondii* or *Besnoitia jellisoni* were resistant to footpad challenge with *M. leprae.* Resistance was manifested by lower numbers of recoverable *M. leprae* in the footpads of protozoal-infected mice and was enhanced in *Toxoplasma*-infected mice by a booster injection of *Toxoplasma* antigen in the infected footpad [11]. However, murine is not a good model to study the leprosy infection, since rodents do not systemically develop leprosy disease. There are no studies about immune response modulation in patients co-infected with *M. leprae* and *T. gondii* and the influence of this immunomodulation on the clinical manifestation of leprosy and toxoplasmosis symptoms. These infections have an opposite protection immune response, where toxoplasmosis majority elicits a Th1 cellular immunity, which induces the production of IL-12, IL-2, IFN-α and TFN-α cytokines; while individuals with the most severe form of leprosy (lepromatous) generally develop humoral immune response (Th2 type) with production of IL-4, IL-5, IL-10 and IL-13, which suppress macrophage activities and stimulate mast cell and B lymphocyte activation.

Additionally, our results also suggest a relationship between leprosy and unfavourable economic circumstances, since drinking water has been determined as the main risk factor for *T. gondii* infection for individuals living under poor sanitary conditions in the same studied area [9].

Although further experiments are necessary to confirm the cellular and molecular mechanisms that underpin the effects of *T. gondii* infection on the leprosy manifestation, studies should also consider the order and timing of the infections with two distinct pathogens, since it can significantly influence the host’s response to the second.

## Conclusions

*T. gondii* infection could have a certain influence on the progression of leprosy or disease pathogenesis. However, an in-depth immunological study, including cytokine dosage, may add elements to support these preliminary results. Greater understanding of how leprosy progression are influenced by concurrent *T. gondii* infection could help the design of more effective treatments to control the spread of this infectious disease.

## Data Availability

The dataset supporting the findings of this article is available from the corresponding author upon request.

## Abbreviations

ABTS: 2,2′-Azino-bis (3-ethylbenzothiazoline-6-sulfonic acid)
BSA: Bovine serum albumin
*CI*: Confidence Interval
CT: Household contacts
DL: Dimorph leprosy
ELISA: Enzyme-linked immunosorbent assay
HC: Healthy controls
HIV: Human Immunodeficiency Virus
HRP: Horseradish peroxidase
IDL: Indeterminate leprosy
IFN: interferon
IgG: Immunoglobulin G
IgM: Immunoglobulin M
IL: Interleukin
LL: Lepromatous leprosy
LP: Leprosy patients
MB: Multibacillary
*OR*: Odd ratio
PB: Paucibacillary
PBS: Phosphate-buffered saline
PBST: Phosphate-Buffered Saline Tween
PGL-1: Phenolic glycolipid-1
SDS: Sodium dodecyl sulfate
STAg: Soluble *Toxoplasma* antigen
TGF: Transforming growth factor
Th: T helper
TL: Tuberculoid leprosy
WHO: World Health Organization
